# Study on the Relationships between Microscopic Cross-Linked Network Structure and Properties of Cyanate Ester Self-Reinforced Composites

**DOI:** 10.3390/polym11060950

**Published:** 2019-06-01

**Authors:** Hongtao Cao, Beijun Liu, Yiwen Ye, Yunfang Liu, Peng Li

**Affiliations:** Research Institute of Carbon Fiber and Composite Materials, Beijing University of Chemical Technology, Beijing 100029, China; 2017200495@mail.buct.edu.cn (H.C.); 2018200278@mail.buct.edu.cn (B.L.); 2018200500@mail.buct.edu.cn (Y.Y.)

**Keywords:** cyanate ester, self-reinforced composite, cross-linked network, mechanical property

## Abstract

Bisphenol A dicyanate (BADCy) resin microparticles were prepared by precipitation polymerization synthesis and were homogeneously dispersed in a BADCy prepolymer matrix to prepare a BADCy self-reinforced composites. The active functional groups of the BADCy resin microparticles were characterized by Fourier transform infrared (FT-IR) spectroscopy. The results of an FT-IR curve showed that the BADCy resin microparticles had a triazine ring functional group and also had an active reactive group -OCN, which can initiate a reaction with the matrix. The structure of the BADCy resin microparticles was characterized by scanning electron microscopy (SEM) and transmission electron microscopy (TEM). From the TEM results, the BADCy resin microparticles dispersed in the solvent were nano-sized and distributed at 40–60 nm. However, from the SEM results, agglomeration occurred after drying, the BADCy resin particels were micron-sized and distributed between 0.3 μm and 0.6 μm. The BADCy resin prepolymer was synthesized in our laboratory. A BADCy self-reinforced composite was prepared by using BADCy resin microparticles as a reinforcement phase. This corresponds to a composite in which the matrix and reinforcement phase are made from different morphologies of the same monomer. The DSC curve showed that the heat flow of the microparticles is different from the matrix during the curing reaction, this means the cured materials should be a microscopic two-phase structure. The added BADCy resin microparticles as reaction sites induced the formation of a more complete and regular cured polymer structure, optimizing the cross-linked network as well as increasing the interplay between the BADCy resin microparticles and prepolymer matrix. Relative to the neat BADCy resin material, the tensile strength, flexural strength, compressive strength and impact strength increased by 98.1%, 40.2%, 27.4%, and 85.4%, respectively. A particle toughening mechanism can be used to explain the improvement of toughness. The reduction in the dielectric constant showed that the cross-linked network of the self-reinforced composite was more symmetrical and less polar than the neat resin material, which supports the enhanced mechanical properties of the self-reinforced composite. In addition, the thermal behavior of the self-reinforced composite was characterized by thermogravimetric analysis (TGA) and dynamic mechanical thermal analysis (DMTA). The results of DMTA also establishes a basis for enhancing mechanical properties of the self-reinforced composite.

## 1. Introduction

The concept of self-reinforcement was first proposed in 1975 by Capiati and Porter in their study of PE/PE fiber composites [1]. At present, self-reinforcement mainly refers to the use of special physical forming methods for controlling polymer morphology. The basic concept of self-reinforced materials is the creation of a one-, two- or three-dimensional alignment within the matrix to fulfill the role of matrix reinforcement [2]. For self-reinforcement composites in which the reinforced phase and matrix have different microstructure but belong to the same family of polymers, the two phases are fully compatible. There are no interface problems between the reinforced phase and the matrix [3,4]. The reinforcement phase is required to have higher stiffness and strength than the matrix so that the stress can be transferred from the “weak” matrix to the “strong” reinforced structure. Based on the balance between performance and cost, self-reinforced materials can compete with traditional composite materials in various fields.

A thermosetting resin is a polymer that is irreversibly hardened by curing from a soft solid or viscous liquid prepolymer or resin [5]. Curing is induced by heat or suitable radiation and may be promoted by high pressure or with a catalyst [6]. Latest research also include using magnetic nanoparticles [7] and ionic liquid [8]. It results in chemical reactions that create extensive cross-linked between polymer chains to produce an infusible and insoluble polymer network that imparts properties such as electrical insulation, corrosion resistance and high-temperature resistance to composite materials. Typical examples of such networks are epoxy (EP) resin, phenolic resin, cyanate ester (CE) resin and unsaturated polyester resin. These materials are widely used in the construction industry [9], the chemical engineering [10], the electronic industry [11], and the aerospace industry [12]. However, the most prominent disadvantage of the thermosetting resin is its brittleness after curing, which limits its use in certain areas.

A common modification method is to prepare composite materials by using inorganic particles [13,14], elastomers [15,16], hyperbranched polymers [17,18], thermoplastic resins [19] and crystal polymers [20]. Due to the different phases of the reinforced phase and matrix, defects caused by interface problems have always been the focus of composite research [21]. If the compatibility of the two materials is poor, then there will be many microdefects in the composite materials, resulting in a substantial decrease in the final performance. Although interface problems can be addressed by functional group modification, the process is cumbersome. Besides, due to the different coefficient of thermal expansion of the reinforcement phase and matrix, stress concentration occurs during the curing process. It is worth noting that the above methods may sacrifice some of the excellent properties of the matrix resin.

**Figure 1:**
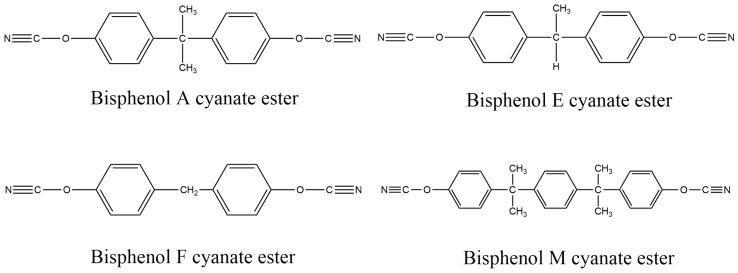
Chemical structures of different dicyanate ester monomers.

CE resins are novel high-performance thermosetting resins similar to EP and bismaleimide (BMI) resins. CE monomers have a variety of chemical structures, and their properties are related to the type of monomer used [22]. Bisphenol A cyanate ester is the main product, although bisphenol E, bisphenol F and bisphenol M are also used. The chemical structure of several monomers is shown in Figure 1. CE resin comprises large molecules with a high cross-linked density network structure containing a triazine ring. This structure of CE resin has demonstrated many unique properties, such as a high glass transition temperature, easy processability, low contractibility rate, low water absorption, ultralow dielectric constant (ε of 2.8–3.2) and dielectric loss (tanδ of 0.002–0.008) values comparable with those of EP, BMI, and polyimide (PI) resins. Because of the combination of a series of advantages of other thermosetting resins, CE resin has broad application prospects.

The purpose of the present study is to prepare a self-reinforced material, taking bisphenol A dicyanate (BADCy) ester as an example. We innovatively used BADCy monomer to prepare BADCy resin microparticles by precipitation polymerization synthesis. BADCy self-reinforced composite materials were prepared by using the BADCy resin microparticles as the reinforced phase. Finally, the relationships between cross-linked network and performance were analyzed.

## 2. Materials and Methods

### 2.1. Materials

BADCy monomer was purchased from Yangzhou Techia Material Co., Ltd. (Yangzhou, China) Zinc acetylacetonate hydrate, nonylphenol and xylene were purchased from Aladdin (Shanghai, China) and Macklin (Shanghai, China), respectively. All chemicals were used as received.

### 2.2. Preparation of BADCy Resin Microparticle

A certain proportion of xylene and BADCy monomer (The mass ratio of solvent to solute is 100:5) was added to a three-necked flask and stirred (100 °C) until the BADCy monomer completely dissolved. Then, zinc acetylacetonate hydrate and nonylphenol were added as catalysts that were dissolved in advance (Each is two thousandths of the mass of the monomer). The mixture was heated at 100 °C and stirred for 1 h. The temperature was raised to 130 °C, and the reaction mixture was allowed to continue to react for 3.5 h. The BADCy resin microparticles were obtained after centrifugation, washing and vacuum drying.

### 2.3. Preparation of BADCy Prepolymer Resin

First, the BADCy monomer was melted at 100 °C. Second, moisture was removed under vacuum. Finally, the liquid was heated at 200 °C for 2 h, then the temperature was cooled to 180 °C, and heating was continued for 8 h until a clear homogeneous melt was obtained. The viscous melt was defined as the BADCy resin prepolymer.

### 2.4. Preparation of Self-Reinforced Composite Materials

The prepared BADCy resin microparticles were added to the prepolymer in different mass percentages. The mixture was stirred at 130 °C for 1 h, degassed at 100 °C for 30 min, and then cured via the following procedure: 180 °C/2 h + 200 °C/2 h + 220 °C/2 h.

### 2.5. Characterization and Measurements

FT-IR spectra was recorded on a Nicolet 8700 FT-IR spectrometer (Thermo Fisher Scientific, Shanghai, China) in transmittance mode. A wavenumber range of 400–4000 cm^−1^, a resolution of 4 cm^−1^, 32 scans, and the KBr tablet method were used.

Scanning electron microscopy (SEM) was carried out using an S-4700 cold-field scanning electron microscope (Hitachi, Japan). The accelerating voltage was 20 kV, and the samples were coated with gold.

Transmission electron microscopy (TEM) was performed with an HT7700 biological transmission electron microscope (Hitachi, Japan). A stable dispersion of BADCy resin microparticles was prepared by ultrasonic treatment. A drop of this stable dispersion was added onto a copper grid and dried.

Differential scanning calorimeter (DSC) was performed on a TA Instruments DSC25 calorimeter (TA Instruments, New Castle, DE, USA) at a heating rate of 10 °C/min, a N_2_ flow of 50 mL/min, and a temperature range of 50–380 °C.

Thermogravimetric analysis (TGA) was performed on a NETZSCH Geratebau Gmbh STA449F5 thermogravimetric analyzer (NETZSCH, Bavaria, Germany) at a heating rate of 10 °C/min, a N_2_ flow of 50 mL/min, and a temperature range of 0–800 °C.

Dynamic mechanical thermal analysis (DMTA) was performed with a type of DMTA-V instrument from American Rheological Science Corporation (Piscataway, NJ, USA). A single cantilever clamp was used under the following conditions: temperature range of 50–280 °C, frequency of 1 Hz and heating rate of 5 °C/min.

The mechanical properties of the composites were recorded on an INSTRON-1121 universal testing machine (Instron Corporation, Norwood, UK). The specimens were tested according to Chinese national standards GB/T 2567–2008.

The dielectric constant and dielectric loss of the composites were tested with an Agilent 4294A precision impedance analyzer (Agilent Technologies Inc., Santa Clara, CA, USA) with a frequency range of 10^4^–10^6^ at 25 °C.

## 3. Results and Discussion

### 3.1. Characterization of BADCy Resin Microparticles

**Figure 2:**
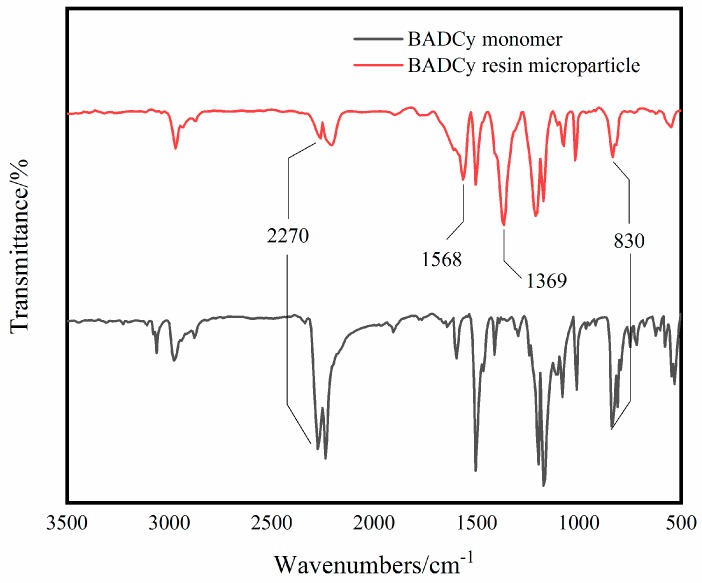
FT-IR spectra of the BADCy monomer and BADCy resin microparticles.

The FT-IR spectra curves of the BADCy monomer and BADCy resin microparticles are shown in Figure 2, and FT-IR data of the main chemical groups in the structures are shown in Table 1. In the FT-IR spectra curve of the BADCy resin microparticles, the new peaks at 1568 cm^−1^ and 1369 cm^−1^ can be assigned to the triazine ring functional groups.

**Figure 3:**
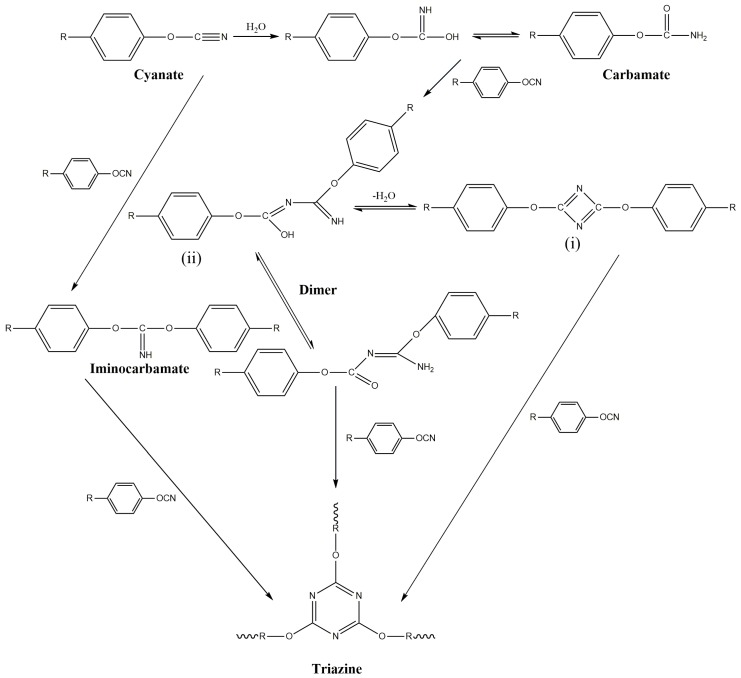
The self-polymerization mechanism of the CE monomer.

**Figure 4:**
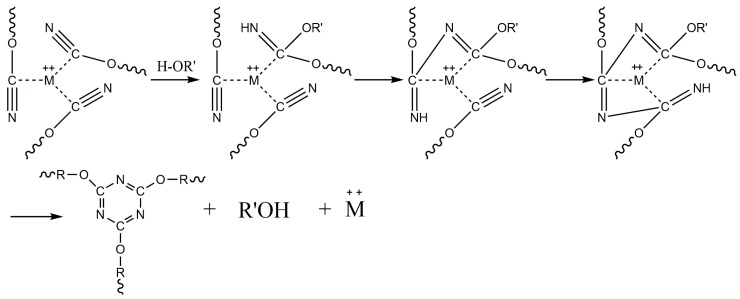
A coordination catalysis model for the polymerization of CE monomers.

CEs are a class of compounds in which the hydrogen atom of a phenolic -OH group is substituted by a cyanide group [23]. The resulting product with an -OCN group is named a CE. CE monomers can be converted in a controlled manner into CE prepolymer resin by self-polymerization [24]. The chemistry of the reaction is a trimerization of three -OCN groups into a triazine ring. This reaction can be carried out by heating, either alone at an elevated temperature or at a lower temperature in the presence of a suitable catalyst [25]. The most common catalysts are transition metal complexes of cobalt [26], copper [27], manganese [28], chromium [29], iron [30], tin [31] and zinc [32]. Active hydrogen compounds such as phenols, amines and imidazole are considered cocatalysts [26,27]. The self-polymerization mechanism of the CE monomer and the catalytic effect of the catalyst are shown in Figure 3 and Figure 4 [24,33], respectively.

Based on the peak at 1568 cm^−1^ and 1369 cm^−1^, BADCy resin microparticles were prepared from the BADCy monomer by self-polymerization. In addition, compared to that of the BADCy monomer, the -OCN absorption peak at 2270 cm^−1^ of the BADCy resin microparticles is reduced. This result is significant and will be discussed later.

**Figure 5:**
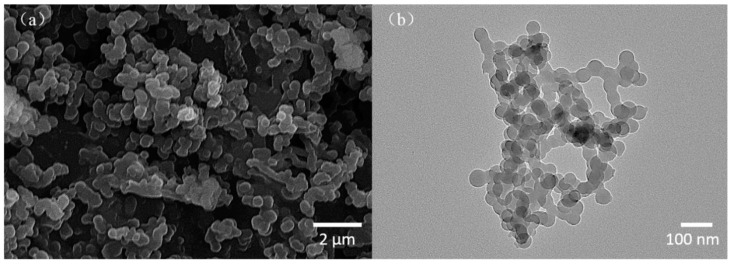
Typical SEM (**a**) and TEM (**b**) images of BADCy resin microparticles.

SEM and TEM images of BADCy resin microparticles were shown in Figure 5a,b, respectively. From the SEM images of BADCy resin microparticles, a large quantity of aggregates (micron-sized) were observed. A single aggregation particle ranged in size from 0.3 μm to 0.6 μm. TEM showed that aggregation was diminished in xylene solution. As shown in Figure 5b, the microparticles were better dispersed, and a large aggregation phenomenon was rarely observed. Moreover, the size distribution of the microparticles became more homogeneous. Microparticles with diameters ranging from 40 nm to 60 nm were observed (nanometers).

The occurrence of the aggregation phenomenon is attributed to the drying process. During the heating process, as the solvent evaporates, the nanoparticles tend to aggregate, and when a certain size is reached, a stable structure is achieved. In addition, no surfactant or dispersant was added throughout the entire preparation process (if the surfactant or dispersant is not completely removed, it will affect the material properties), and nanoparticles would aggregate more due to their higher surface energy than microparticles. In conclusion, the resulting dry powders are micron-sized.

### 3.2. Mechanical Properties of Self-Reinforced Composites

**Figure 6:**
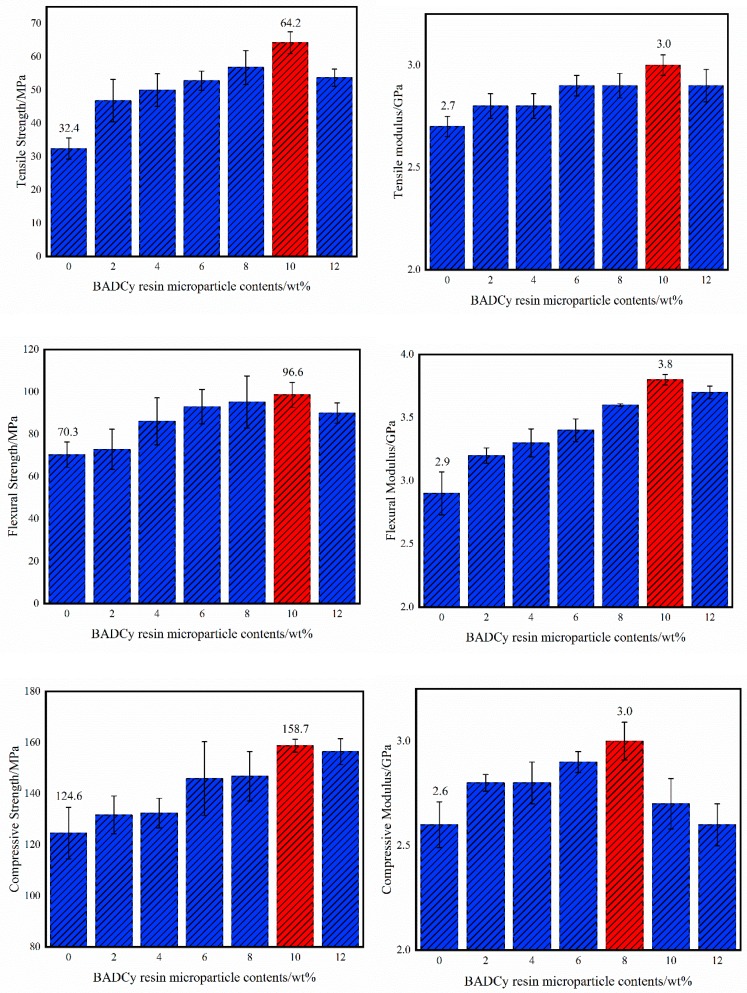
Mechanical properties of the BADCy resin self-reinforced composites with different BADCy resin microparticle contents.

The mechanical properties of the self-reinforced composites with different BADCy resin microparticle contents are shown in Figure 6. Compared with those of neat BADCy materials, the mechanical properties of the self-reinforced composites are improved. Detailed data are shown in Table 2.

When the content of BADCy resin microparticles is 10%, the tensile strength of the self-reinforced composite reaches a maximum of 64.2 MPa, the tensile modulus reaches a maximum of 3.0 GPa, the flexural strength reaches a maximum of 98.6 MPa, the flexural modulus reaches a maximum of 3.8 GPa, and the compressive strength reaches a maximum of 158.7, corresponding to 98.1%, 11.1%, 40.2%, 31.0% and 27.4% increases, respectively. When the content of BADCy resin microparticles is 8%, the compressive modulus reaches a maximum of 3.0 GPa, increasing by 15.4%.

**Figure 7:**
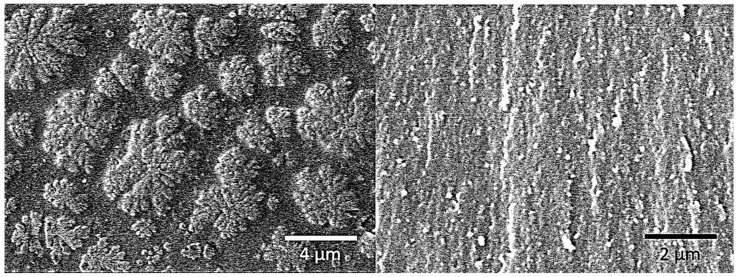
SEM micrographs of the fracture surface of a vinyl ester resin.

The improvement in the strength could be related to a more regular and complete cross-linked network. Curing is a chemical process that induces the toughening or hardening of a polymer material via cross-linked of polymer chains [6]. In the process of cross-linked polymer chains, many molecular aggregates called gel particles exist. The gel particles are stably dispersed in the low molecular weight oligomer. As the reaction progresses, the number of microgel particles increases, the volume becomes larger, and the particles collide with each other, resulting in an increase in the viscosity of the system. Finally, the initial phase is surrounded by a new phase, and the gel phase becomes a continuous phase [34]. The previous study have reported the morphology and size of the microgel particles formed during the curing process of the resin [35]. As is shown in Figure 7, the clusters of the microgels disperse throughout the entire fractured surface, each cluster of microgels contains many microgels with coral-like structures. In fact, the microgel clusters observed at this time were the result of the growth of the microgel particles. 

**Figure 8:**
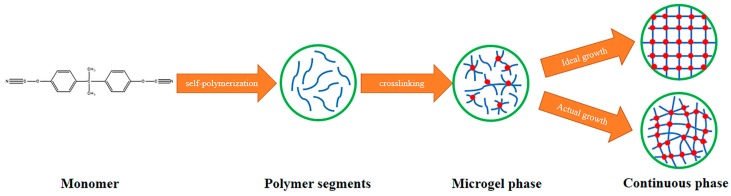
Comparison of cross-linked networks formed under ideal and actual conditions.

However, the formation and distribution of the gel particles are random. Only in an ideal situation can an absolutely perfect cross-linked network structure be formed, whereas the actual situation is complicated. Figure 8 is a schematic view showing the formation of polymer segments and curing using BADCy monomer as an example.

**Figure 9:**
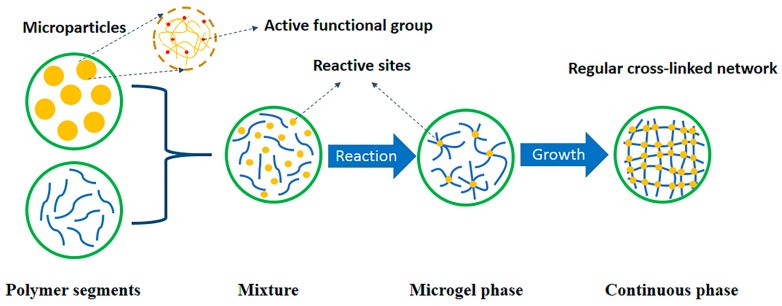
A schematic diagram of the formation of the self-reinforced composites in which the added microparticles are capable of initiating a curing reaction, resulting in a higher degree of regularity in the formed cross-linked network.

Due to the more complete structure of a cross-linked network formed by curing, the resulting material has better application performance. Thus, the added BADCy resin microparticles are equivalent to the microgel particles, and their function is to serve as a reaction site, inducing the curing of the BADCy prepolymer to form a more complete and regular cross-linked network structure and thereby markedly improving the performance. This process is shown in Figure 9.

In Figure 1, the prepared BADCy resin microparticles still have a peak at 2270 cm^−1^, which indicates that the BADCy resin microparticles have functional groups -OCN and can continue to react with the matrix. In the curing process, in addition to the reaction between the microparticles and the matrix, the resin matrix also undergoes a process of gel particle formation. Since the formation of microgel particles requires a large amount of energy, and the reaction between the microparticles and the matrix is relatively easy, it is macroscopically expressed as a reaction between the particles and the matrix. Therefore, the BADCy resin microparticles can serve as reaction sites.

**Figure 10:**
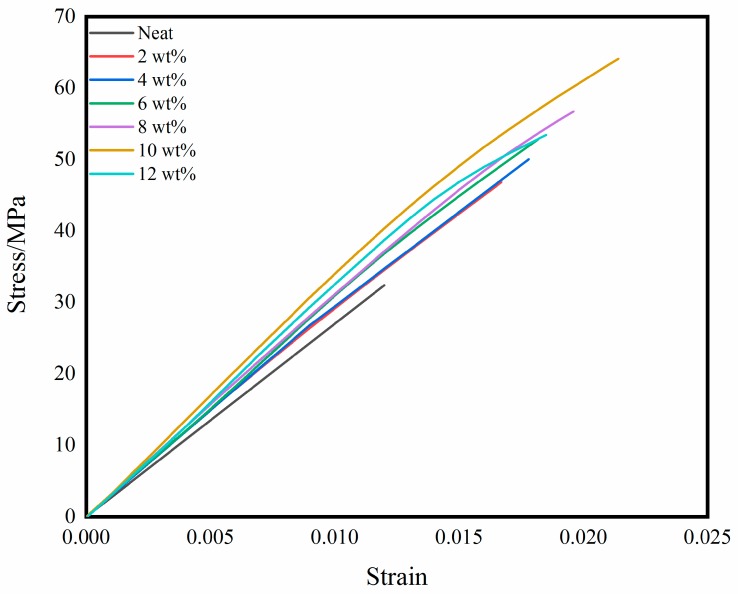
Comparison of measured stress-strain curves for the different samples.

The representative stress-strain curves are shown in Figure 10. Since the CE resin is a brittle material, the curves approximately increase linearly at room temperature until samples break. And the fracture occurs before the yield point occurs. The difference is that the curve of each system is different from the area enclosed by the X axis, that is, the fracture energy is significantly different. The fracture energy of self-reinforced composites is significantly higher than that of neat resin. From a practical point of view, the fracture energy is the basis of the impact test. Since the stress-strain curve is not easy to measure in the impact test, the impact strength is conventionally expressed by the fracture energy of the standard sample.

**Figure 11:**
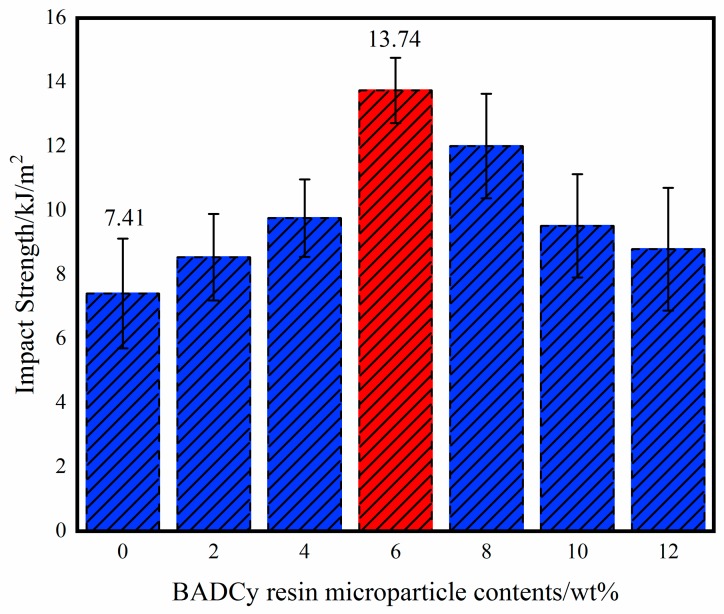
Impact strength of the BADCy resin self-reinforced composites with the different BADCy resin microparticle contents.

Figure 11 shows the impact strength of the composite. Impact strength is a simple and effective method to characterize toughness. When the content of BADCy resin microparticles is 6%, the impact strength reaches a maximum of 13.74 kJ/m^2^. Compared with that of the neat BADCy resin material, the impact strength of the self-reinforced composites was increased by 85.4%. Therefore, not only the strength but also the toughness of the self-reinforced composites is markedly improved.

**Figure 12:**
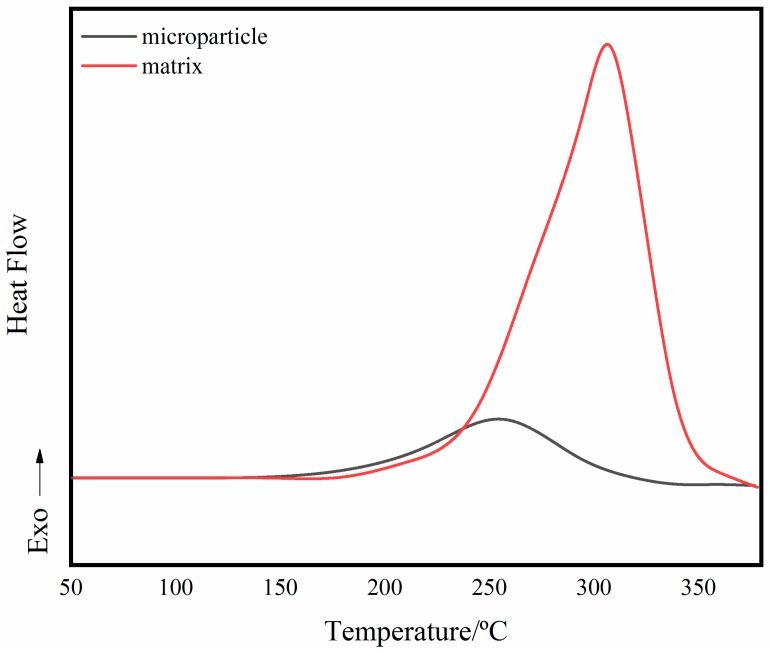
The DSC curves of the BADCy resin microparticles and the matrix.

The improvement of toughness can also be explained by particle toughening [36]. From the result of the DSC curves (Figure 12), the prepared BADCy resin microparticles are still exothermic during the heating process. It has been known from FT-IR results that the surface of the BADCy resin microparticles still have reactive functional groups. The heat flow is due to the curing reaction, which indicates that the prepared microparticles are reactive. However, the heat release amount is significantly reduced compared with the resin matrix, that is, the degree of curing of the microparticles is much larger than that of the resin matrix. This allows the cured materials to have a two-phase structure at the microscopic level. The presence of the BADCy resin microparticles can prevent crack propagation or passivation [15,37]. And the surface of the microparticle still has -OCN functional groups, which cause no defects between the matrix and the microparticles due to interfacial problems.

**Figure 13:**
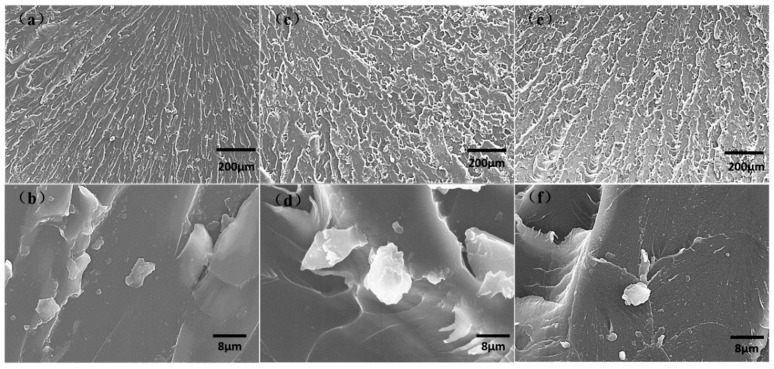
SEM fractographs of the neat BADCy resin and self-reinforced composites: (**a**,**b**) neat resin; (**c**,**d**) composite containing 6 wt% BADCy resin microparticles and (**e**,**f**) composite containing 10 wt% BADCy resin microparticles.

Representative SEM images of the impact fraction surface of the composites with different BADCy resin microparticle contents are shown in Figure 13a–f represent the neat resin, the composite containing 6 wt% microparticles and the composite containing 10 wt% microparticles, respectively. The fracture surface of the BADCy matrix composite exhibits a river shape, reflecting that the matrix facture is a typical brittle mode fracture [38]. It can be seen that with increasing BADCy resin microparticle content, the fracture surface becomes rougher and more irregular, and plastic deformation occurs. Meanwhile, with increasing BADCy resin microparticle loading, the strength of the interaction between the microparticles and the matrix is enhanced. The increased covalent bond strength between the particles and the matrix provides great resistance to cracks, which results in increased composite toughness. Moreover, many open-cell regions can be clearly observed in Figure 13c,d. These open-cell regions could absorb the fracture and hinder crack propagation. Large separated clusters due to the aggregation of BADCy resin microparticles at 10% content are also observed in Figure 13f. This aggregation occurs because when the concentration of the microparticles further increases and exceeds a certain limit, the microparticles are close enough to each other to be easily agglomerated, and the material is more susceptible to plastic deformation when subjected to impact, developing into macroscale stress corrosion cracking. This change would result in more rapid crack initiation and impact failure. Therefore, the impact strength of the reinforced composite materials cannot be improved much by incorporating high BADCy resin microparticle contents.

### 3.3. Thermal Properties of Self-Reinforced Composites

**Figure 14:**
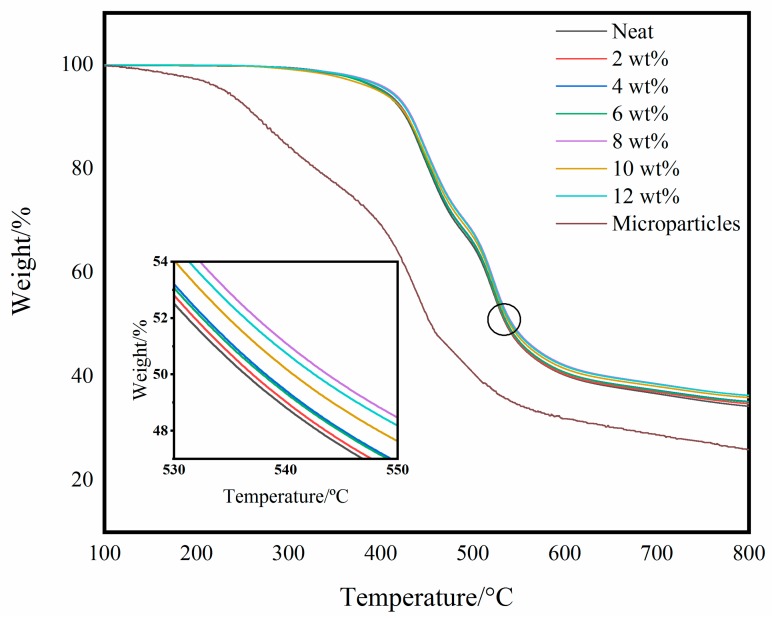
TGA curves of the neat resin, self-reinforced composites and microparticles.

TGA curves of BADCy self-reinforced composites with various contents of BADCy resin microparticles are shown in Figure 14. The temperature at the 5%, 10%, 20% and 50% weight loss are shown in Table 3.

It can be seen that the weight loss of all samples before 300 °C is not obvious. Marked weight loss occurs between 400 °C and 600 °C. This is mainly caused by the degradation of polymer chains. The temperature with weight loss of 5% was defined as initial degradation temperature (IDT), and the temperature with weight loss of 10% was also compared. From the results of Table 3, the IDT of the self-reinforced materials are higher than the neat sample and the sample with the BADCy resin microparticle content of 8 wt% reaches the maximum. When the weight loss of all samples is 10%, the trend of temperature changes is basically consistent with the IDT. The inset curve is the weight loss around 50%. It can be seen that the self-reinforced composite containing 8 wt% BADCy resin microparticles still has a high thermal decomposition temperature. The above results indicate that the self-reinforced materials have more excellent thermal stability. On the other hand, the TGA curve of the BADCy resin microparticles was also shown in Figure 14. Since the prepared BADCy resin microparticles have a small molecular weight, the decomposition temperature to the specified quality is always lower than the neat resin and self-reinforced composites. Related study reported that the improvement of the thermal stability of nanocomposites was due to the higher thermal stability of the nanoparticles than the matrix, resulting in an increase of nanocomposites [39]. However, it is can be seen that the improvement of thermal stability of the self-reinforced composites is related to other factors. The early study on nanocomposites emphasized that the particle-matrix interfical interaction has been suggested to responsible for the improved thermal stability in nanoparticles [40,41]. For the self-reinforced composites, covalent bonding between microparticles and matrix markedly reduced the segmental motion and the diffusion of the decomposition products. In Table 3, the char yield at 800 °C is increasing with the increased BADCy resin microparticles. This phenomenon is due to the decreasing free volume fractions in the systems [42]. The increasing char yield of the self-reinforced composites can be attributed to their limited segmental motion and low chain scission rate, which results in the formation of a mount of char yield due to decreased loss of the volatile products.

**Figure 15:**
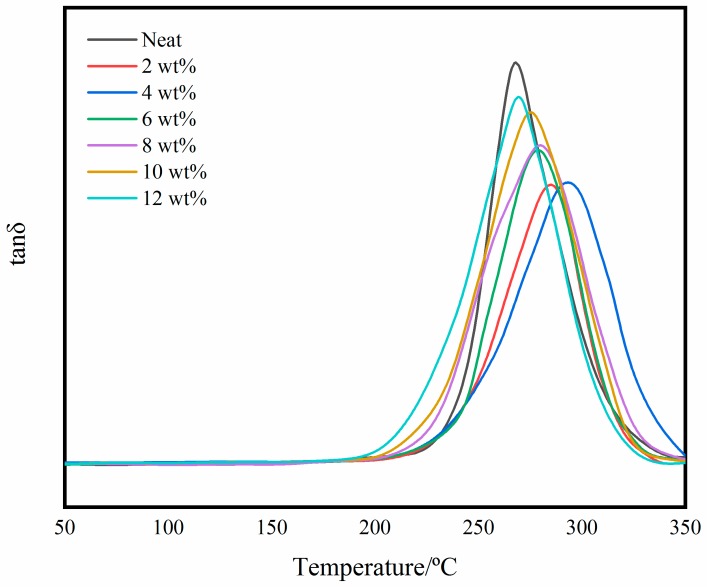
DMTA loss factor curves of the neat resin and the self-reinforced composites.

Figure 15 is a graph of the DMTA loss factor. The temperature corresponding to the peak is the glass transition temperature. It can be seen that the glass transition temperature is 268 °C, 285 °C, 293 °C, 279 °C, 280 °C, 276 °C and 269 °C, respectively. It can be concluded that the glass transition temperature of the self-reinforced composites improves compared with neat matrix. In addition, the reduction in peak loss also reflects the increase in the toughness of the self-reinforced composites.

Microparticles networks and interfacial interactions are two primary factors which dominate the mechanical properties [43]. Some experiments showed that interfacial interactions had a large impact on the mechanical properties of nanocomposites [42]. The peak value of *T*_g_ decreases significantly with the microparticles loading. Studies have shown that the peak height of *T*_g_ were inversely proportional to the volume fraction of confined segments in the interface layers [44]. It can be concluded that the added BADCy resin microparticles increase the covalent bonding between the microparticles and the matrix. This is also reflected in the change in the width of peaks. The relaxation of segments at different locations in the system will span a larger temperature range with BADCy resin microparticles increase. The segments near the microparticles will experience greater confinement, therefore their relaxation occurs at higher temperatures. Meanwhile, the increase in *T*_g_ is also related to the increased cross-linked network. In contrast, a larger loading will make the *T*_g_ improvement less obvious, perhaps because the microparticle density is larger and aggregation occurs. In summary, the change in *T*_g_ is closely related to the dispersion state of the microparticles and the interfacial effects.

### 3.4. Dielectric Properties of Self-Reinforced Composites

**Figure 16:**
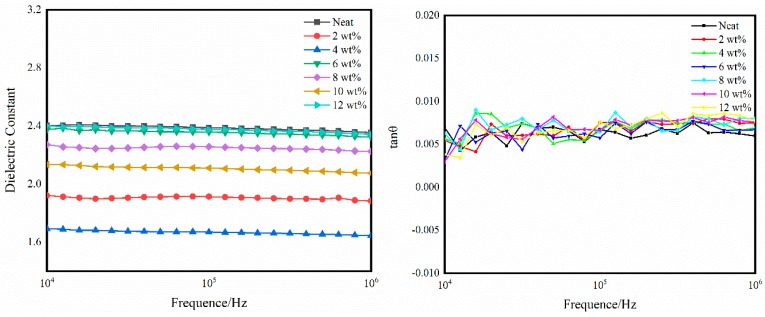
Dielectric properties of the neat resin and self-reinforced composites.

Figure 16 shows the dielectric constant and dielectric loss of the self-reinforced composite. It can be concluded that as the particle content increases, the dielectric constant decreases first and then rises. However, there is basically no significant change in dielectric loss (the dielectric loss of a CE resin is usually 0.002–0.008). Importantly, the dielectric constant reaches a minimum value of 1.7 when the BADCy resin microparticle content is 4 wt% (the dielectric constant of a CE resin is usually 2.8–3.2).

CE resin has a remarkably low dielectric constant, mainly because of the existence of a highly symmetrical triazine ring structure [45]. The dielectric constant mainly depends on the polarity of the polymer molecule and the density of polar functional groups [46]. The higher the molecular polarity and the polar group density are, the larger the dielectric constant of the material. This conclusion can be verified by the Clausius-Mossotti theory [47].

(1) $$\frac{\varepsilon_{r}-1}{\varepsilon_{r}+2}=\frac{N\alpha }{3\varepsilon_{o}}$$

where ε*_r_*, ε*_o_*, *N* and *α* are the dielectric constant, permittivity of vacuum, number of polarized particles per unit volume and degree of polarization, respectively. The decrease in the dielectric constant may be due to a decrease in the polarizability of the molecules or a decrease in the number of polarized units per unit volume.

The dielectric constant of the composite materials is generally lower than that of the neat resin material, which indicates that the added particles do have a function of enhancing the cross-linked network structure [48]. This result is consistent with the conclusion drawn from the mechanical property experiments.

## 4. Conclusions

The design and optimization of a thermosetting polymer self-reinforced composite require a basic understanding of the structure/property relationship. The BADCy resin microparticle surface affords active functional groups, which permits the systematic study of the evolution of the microstructure and correlation with the behavior of the bulk composite. To establish such a correlation, we prepared BADCy self-reinforced composites with various BADCy resin microparticle loadings. FT-IR spectroscopy results show that the semi-cured BADCy resin microparticles still have reactive functional groups and can initiate self-polymerization as reaction sites. From the SEM images of the impact fraction surface, the microparticles and matrix become continuous phases, and no aggregation occurs. This behavior is probably because the microparticles and matrix belong to the same polymer family, so the compatibility between them is good.

The added microparticles make the cross-linked network more perfect. The strength, modulus and toughness of the self-reinforced composites increased to varying degrees. The improvements in tensile strength and impact strength were more obvious, increasing by 98.1% and 85.4%, respectively. Due to the degree of crosslinking of the microparticles and the matrix are different, resulting self-reinforced composites are in a microscopic two-phase structure. The stress transfer efficiency increased because of the reaction between the microparticles and the matrix. And the presence of microparticles hinders the crack propagation. A more regular cross-linked network structure is also verified by a reduction in the dielectric constant and the Clausius-Mossotti theory. However, the increases in these properties at high microparticle loadings were inconspicuous because of the structure between the microparticles and the matrix was changed at high microparticle loadings. In addition, the DMTA results showed that the addition of microparticles did considerably change the glass transition temperature. And from the results of the TGA curves, it is can be seen that the thermal stability of the self-reinforced composites are improved slightly compared with the neat resin.

## Figures and Tables

**Table 1:** FT-IR bands of the chemical groups in the CE structure.

Chemical Group	-OCN	Triazine Ring	Benzene Ring
Wavenumber/cm^−1^	2270	1568, 1369	860

**Table 2:** Mechanical property summary.

Contents	Neat	2 wt%	4 wt%	6 wt%	8 wt%	10 wt%	12 wt%	Increase
Tensile strength/MPa	32.4	46.8	50.0	52.8	56.8	64.2	53.7	98.1%
Tensile modulus/GPa	2.7	2.8	2.8	2.9	2.9	3.0	2.9	11.1%
Flexural strength/MPa	70.3	72.8	86.1	94.0	95.2	98.6	90.7	40.2%
Flexural modulus/GPa	2.9	3.2	3.3	3.4	3.6	3.8	3.7	31.0%
Compressive strength/MPa	124.6	131.7	132.4	145.9	146.8	158.7	156.4	27.4%
Compressive modulus/GPa	2.6	2.8	2.8	2.9	3.0	2.7	2.6	15.4%
Impact strength/kJ/m^2^	7.41	8.54	9.76	13.74	12.01	9.52	8.79	85.4%

**Table 3:** Temperature at the specified weight loss rate for the self-reinforced composites and char yield at 800 °C.

Contents	Neat	2 wt%	4 wt%	6 wt%	8 wt%	10 wt%	12 wt%
*T*_d5%_ (°C)	400	403	402	401	410	402	408
*T*_d10%_ (°C)	427	429	429	428	433	429	432
Char yield (%)	34.25	34.74	35.17	35.18	36.32	35.93	36.36
